# The accuracy of Infrared sensor detection in a smart toilet

**DOI:** 10.12688/f1000research.73086.2

**Published:** 2022-03-14

**Authors:** Amar Lokman, Kirenraj Rajendran, R Kanesaraj Ramasamy

**Affiliations:** 1 Faculty of Computing and Informatics, Multimedia university, Cyberjaya, Selangor, 63100, Malaysia

**Keywords:** Infrared sensor, Raspberry Pi, Internet of Things (IoT)

## Abstract

**Background:** Infrared (IR) sensors are useful tools for detecting distance and proximity. However, these sensors are not good at detecting edges of an area, therefore when used in a smart toilet it has difficulty in detecting the orientation and position of the user’s body. The aim of this study was to design an IR sensor for a smart toilet with a more accurate and consistent detection.

**Methods:** A total of 12(six men and six women) participants with different body types were involved in this study. IR sensor detection was tested in the sitting and squatting toilets. For the best accuracy, the IR sensor's angle was measured. Red, blue, and red-blue plastic covers were used, as these colors improve precision. The microcontroller was set up to calculate the participant’s distance and presence in the cubicle.

**Results:** Toilet positioning varied greatly depending on whether one is sitting or squatting. For sitting toilet, the red cover was close to the accurate distance at a 172° angle. IR detected a man but not a woman's body. The blue cover provided the same best angle of 172° with a higher sensor distance. When the red and blue cover combination was applied, the reading of 141cm detected both men and women, at 172° angle. The actual distance for squatting toilets was 158cm. The optimal angle for both red and blue covers was 176°, however the sensor distance was greater for the blue cover. Finally, the red and blue cover combination gave a more accurate distance of up to 163cm from the actual reading, when detecting both genders at a normal angle of 76°.

**Conclusion:** The combination of red and blue cover gave the most accurate detection for the squatting and sitting toilets. The best angle for sitting was 172°, and for squatting was 176°.

## Introduction

Technological advancement across the world allows for almost everything to be connected to each other. The Internet of Things (IoT) defines objects that are instilled with sensors and other technologies that can share data with other devices and systems over the internet ^1^.

Smart Toilet is one of the examples of the IoT system. Smart Toilet implementation consists of infrared (IR) sensor which can accurately measure the distance from the height of the toilet. The IR sensor is connected to Raspberry Pi as a microcontroller, also called a tiny computer that has a router for internet connection.

 The SHARP GP2Y0A710K0F is an IR distance sensor with an extra-long range of 100–500 cm, which is incredibly simple to use. As such, this IR sensor is a preferred option for this study compared to ultrasonic sensors.

**Figure 1.**
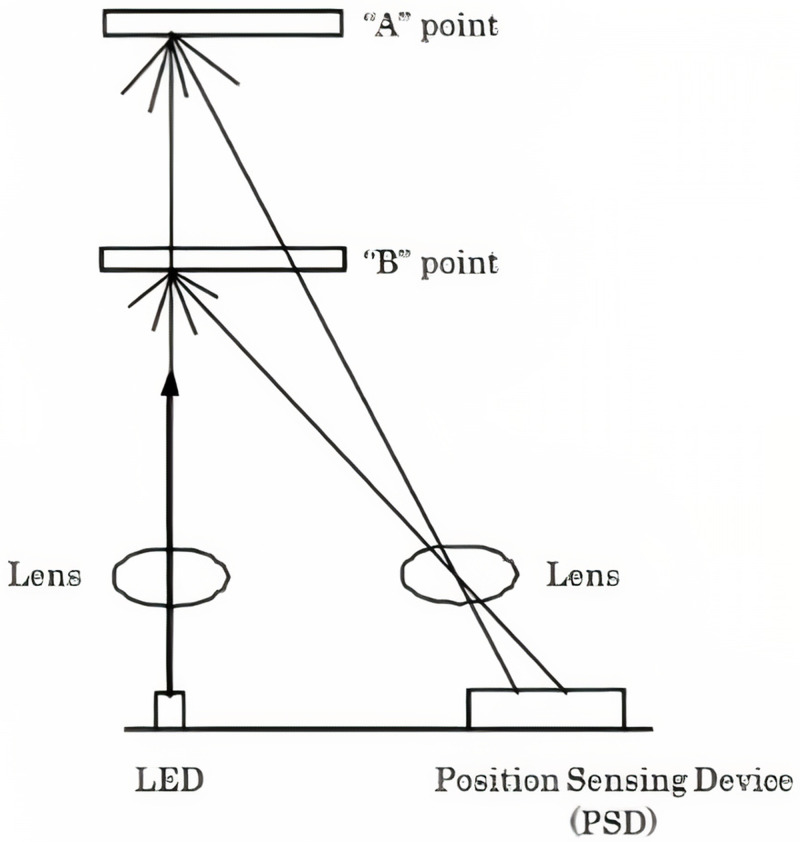
Infrared sensor architecture system ^2^.

The IR sensor measures a distance with the use of the triangulation principle, in which the measurement of the distance is dependent on the angle of the reflected beam. The sensor consists of an IR light-emitting diode (LED) and a Position Sensing Device (PSD) or light detector. The IR emitted from the LED emitter, hits an object which is then mirrored off at a certain angle. This reflected beam will reach the PSD, creating an “optical spot”. As the object's direction/position changes ( Figure 1), the angle of the reflected beam and the direction of the position on the PSD changes as well.

**Figure 2.**
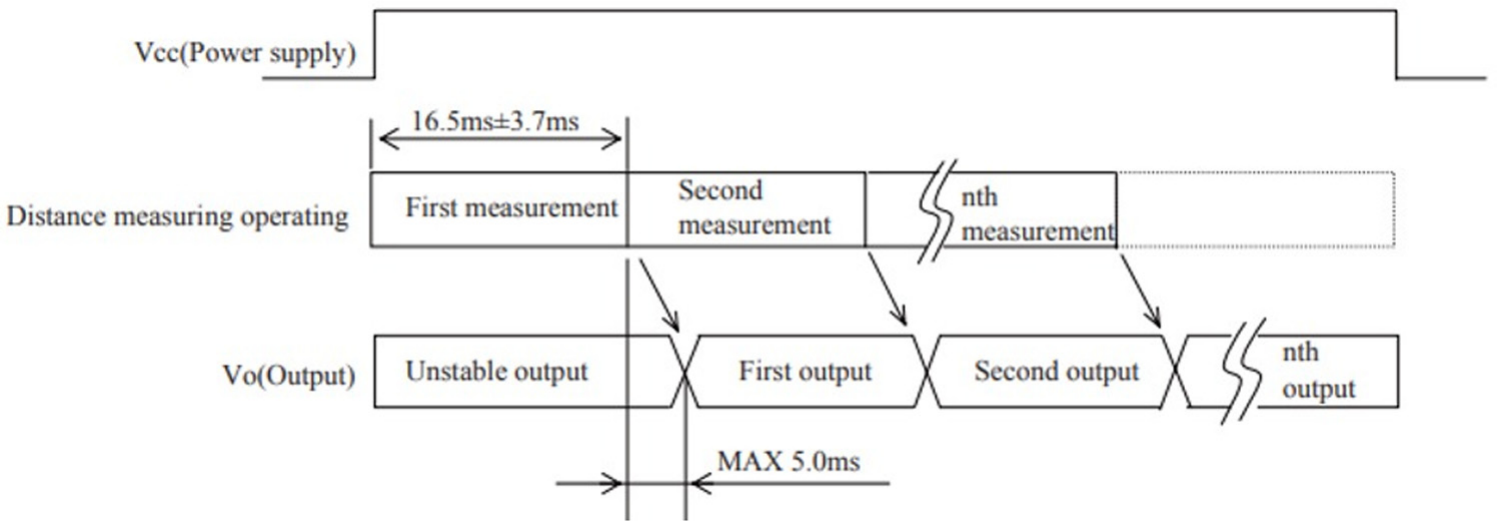
The positioning diagram for measures of distance ^3^.

The sensor has a signal processing circuit that is built-in. This circuit processes the position of the “optical spot” on the PSD to determine the location and the distance of the reflective object. It outputs an analogue signal depending on the distance between the sensor and the object. The output voltage of the SHARP GP2Y0A710K0F ranges from 2.5 - 1.4 V when an object is placed within 100–500 cm distance, respectively ^2^. The input, ground, and analog signals are all connected to three pins on this sensor. To connect to the Raspberry Pi, the MCP 3008 chip is utilized as an analogue to digital converter. The positioning graph for each computation is shown in Figure 2. Each adjustment has a 5ms delay.

**Figure 3.**
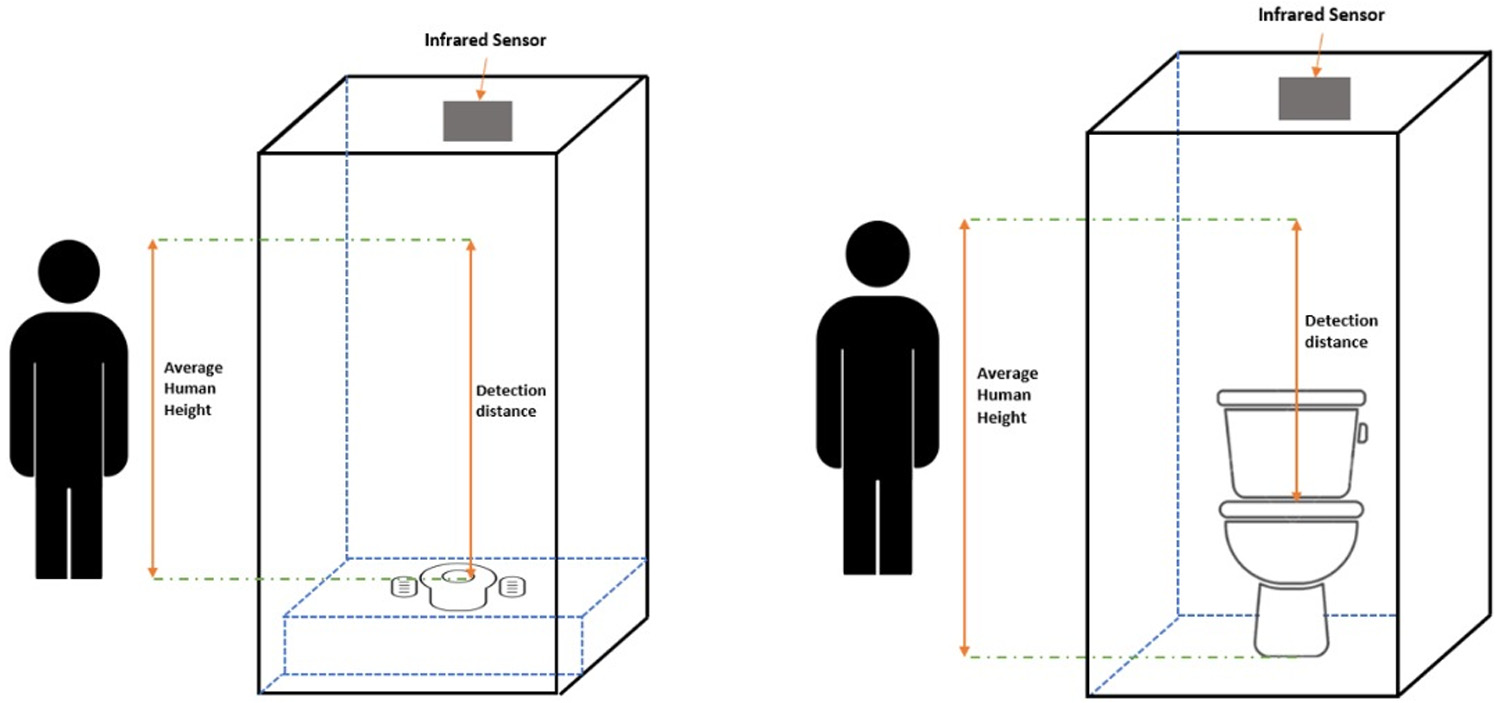
Toilet cubicle for squatting toilet and sitting toilet.

For the IR sensor, detecting the position and the orientation of the user’s body in the toilet is a great challenge, as the sensor responds more accurately to a flat surface compared to a curved one (the position of the participant’s body during sitting or squatting). Additionally, this sensor measures the distance based on the type of toilets used (seated or squatting) ( Figure 3). In this paper, an IR detection system is used to locate people using these two types of toilets. The goal was to ensure sensitivity of the sensor readings for precise distance measurements, and accurate detection of different body shapes and sizes. 

There are many that have used sensors and proven its efficacy ^3^. For example, the complex framework of traffic management ^4^, operates the traffic control system by using IoT, IR sensors, and image processing to make the road system operate efficiently. Car movement and traffic is detected by the IR-sensors. Information on direction of the traffic is then sent to the drivers’ mobile device. The driver uses his/her mobile device to monitor the traffic density and the position of the closest traffic signal. The IR sensor is placed on the roadside pole near the traffic light, where the transmitter and receiver face the road. Disadvantage of this sensor is that it works poorly under the sun as the receiver is sent both the IR waves from the transmitter and the sun causing inaccuracy. Therefore, the IR sensor needs to be installed in a closed box, safe from sunlight and rain.

Singh, A, *et al.* ^5^ suggested a solution based on IoT for reliable and safe collection of waste. The route of waste collection vehicles is dependent on the waste status of the smart bin. In order to optimize the framework upon use, the program uses Cloud Analytics and Deep Learning. This aids with managing the variations in the processing of waste. The rim of the bin has four Infrared Obstacle Line Sensors (the black boxes are the sensors, and the yellow lines are the obstacle routes they cover) mounted on the upper rim of a dustbin. It is possible to mount the sensor system on both lid-based bins and without lid-based bins. A Raspberry Pi 2 board is mounted on the IR sensors. A Wi-Fi Card / Global System for Mobile communication (GSM) module linking it to the internet is mounted on the surface. The board notifies the machine when the dustbin is full. The device is a web application built on Python (Django Framework), which manages all updates from the bins and their exact coordinate positions on a map. The system then plans the strategy and proposes an optimal path.

 Another example is the blind people's assistive IR sensor based smart stick ^6^, which was suggested as a solution for these individuals to detect obstacles on their path. The smart stick uses horizontal and inclined IR sensors, as they are lightweight, inexpensive, have a specific range and have low power consumption compared to ultrasonic sensors. The horizontal IR sensor is located below the hand stick at a height of 90 cm to check the area in front of the blind individual, while the inclined IR sensor is located at a height of 75 cm. The smart stick works by transmitting a pulse of IR signal which travels into the environment. In the absence of an obstacle this signal is not emulated, and as a result no signal, except a dull noise signal, might be sent to the receiver. In the event of finding an obstacle, the signal is transmitted back to the receiver ^6^.

This paper aims to examine how to improve IR sensor for more accurate human detection, and to also assess which cover color will result in more precise measurements.

## Methods

In this study, six men and six women (n=12) participated. We only utilised 12 people because this is a qualitative study to see what angle is optimal for toilets so the sensor can detect them. We used students of varied sizes. Both men and women were included as different body sizes and types affected the reflection of the IR signal. Defecation posture needs to be in the right body position, which is 35° - 45°. The distance of the IR detection for the squatting toilet is greater than the sitting toilet (toilet bowl) ( Figure 3). The average height of male and female participants was taken, to determine the ratio of the body size to the specific area of IR detection. The detection distance ranged from 50 cm to 200 cm.

The SHARP GP2Y0A710K0F IR sensor was placed in an enclosure box to protect the sensor from water damage and other IR radiation, to avoid interference. The enclosure box was then attached to where the cubical celling meets the wall, in both squatting and sitting toilets ( Figure 5). The angle of the IR sensor was measured to obtain the most optimal and accurate distance. The angle of the sensor depended on the plastic covers used; red, blue, and a combination of both covers. Blue and red covers were used as these colours are suitable for infrared sensor detection ^7^, thus these specific colours can provide a more accurate result. The plastic covers were placed directly outside the enclosure box, parallel to the IR sensor emitter and detector, so that the ray can be transmitted from the enclosure box and pass through the plastic cover without an obstacle in between.

**Figure 4.**
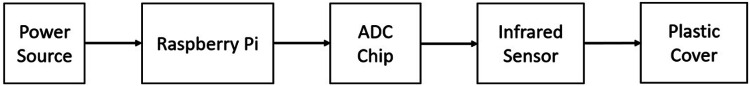
Block diagram of the overall system.

The Raspberry Pi microcontroller was programmed to calculate distance ^8^ and presence of the human body inside the cubicle ( See underlying data) ^9^. IR sensor used analogue voltage input from the Raspberry Pi. MCP3008 chip was used to convert digital output to analogue input for the IR sensor ( Figure 4). This chip has eight output channels, and it connects to the Raspberry Pi by using a Serial Peripheral Interface (SPI) serial connection ^9^. SPI is a protocol for synchronous serial data, that interacts very easily with one or more computers.

**Figure 5.**
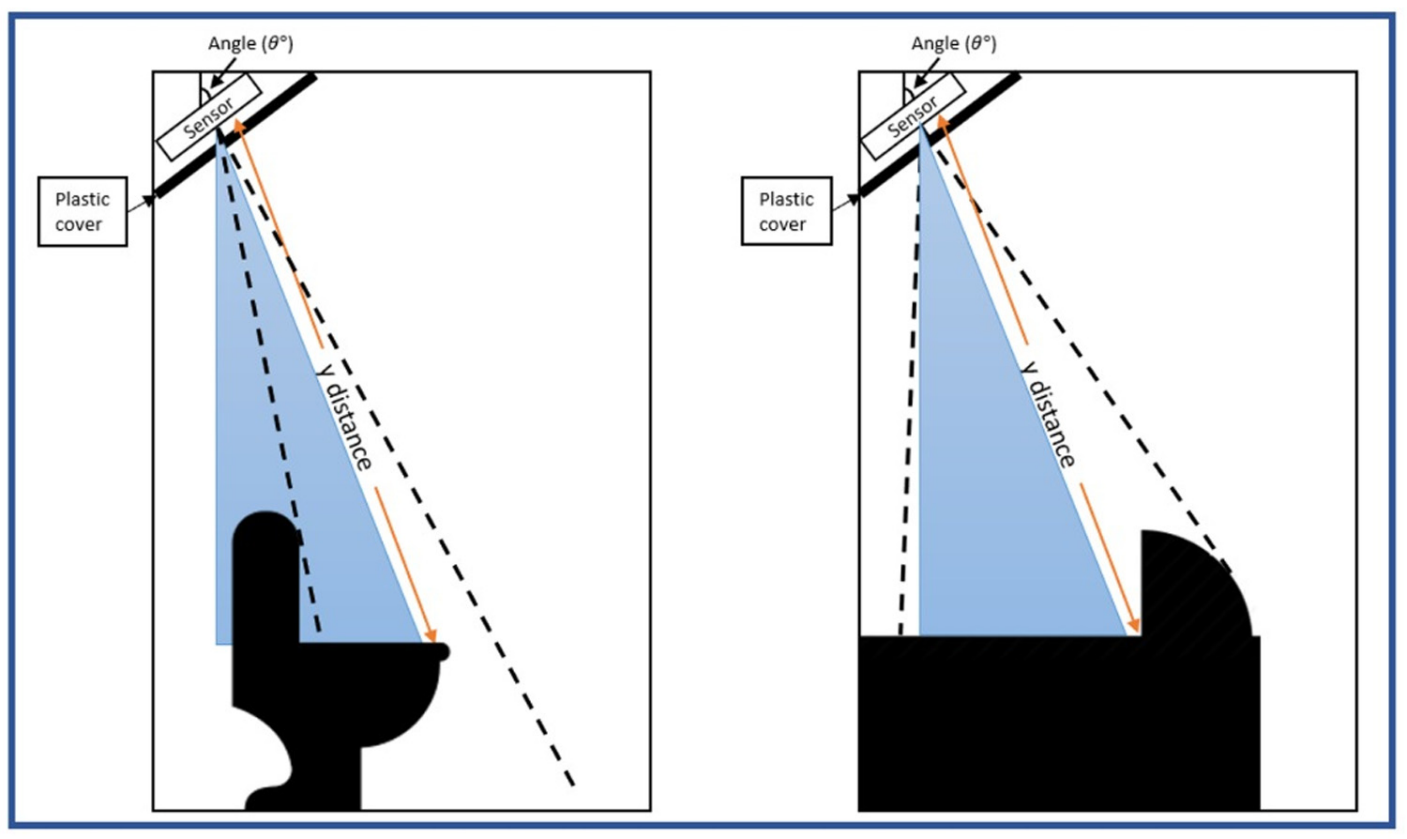
Position of the sensor and plastic cover inside the cubicle.

In general, the sensors were first mounted at a set angle according to Table 1. After that, users must enter one by one. This is done for each colour in turn to get the percentage of correctness. PVC was used as the material. The purpose of utilising this material is to conceal the IR sensor from consumers so that they are not alarmed. Because the IR sensor resembles a camera, we covered it with this material to alleviate user concerns. As IR rays are similar to lights, different colours have varied effects on the IR sensor.

**Table 1.** IR detection for sitting toilet.

Angle ( *θ°*)	Plastic cover	Sensor dist. (cm)	Actual dist. (cm)	Man	Woman	% Error
168	Red Cover	273	141	0	0	93.62
170		248	141	1	0	75.89
172		172	141	1	1	21.99
174		252	141	0	0	78.72
168	Blue Cover	224	141	0	0	58.87
170		214	141	1	0	51.77
172		184	141	1	1	30.50
174		236	141	0	0	67.38
168	Red and Blue Cover	271	141	0	0	92.20
170		263	141	1	0	86.52
172		150	141	1	1	6.38
174		239	141	0	0	69.50

### Raspberry Pi programming function

Raspberry Pi 4 Model B that runs Python as the main language, was used. This model contains a small computer operating on a 4 GB Random Access Memory (RAM) Raspbian operating system, and a built-in WIFI. Overall code is shown in below.


FUNCTION distance ():
   Define analog pin, total distance and avg factor var.
   While average factor <= 75:
       Read analog pin value in voltage unit.
       Distance = 28250 / (voltage – 229.5)
       Total distance = total distance + Distance
   Distance = total distance / average factor
   RETURN distance
   
   


## Results

The result for sitting toilet is shown in Table 1, and result for squatting toilet in Table 2. For sitting toilet, angle stared at 168° to 174° while squatting toilet starts at 174° to 180°. Data 0 and 1 for man and woman indicated the detection of human body. Data 0 represented no detection while data 1 represented detection.

**Table 2.** IR detection for squatting toilet.

Angle ( *θ°*)	Plastic cover	Sensor dist. (cm)	Actual dist. (cm)	Man	Woman	% Error
174	Red Cover	249	158	0	0	57.59
176		189	158	1	1	19.62
178		216	158	1	0	36.71
180		228	158	0	0	44.30
174	Blue Cover	252	158	0	0	59.49
176		175	158	1	1	10.76
178		230	158	1	0	45.57
180		246	158	0	0	55.70
174	Red and Blue Cover	276	158	0	0	74.68
176		163	158	1	1	3.16
178		243	158	1	0	53.80
180		259	158	0	0	63.92

For sitting toilet, red cover received a value close to the actual distance at angle 172°. At angle 170°, IR was able to detect a man, and not a women’s body. This is because at this angle a man’s body which is usually larger in size, is easier to detect than a women’s body. Other angles gave a very high sensor distance, which is not suitable for human body detection. Similarly, the blue cover resulted in the same best angle, which was 172°, however the sensor distance was higher than the red cover. The red and blue cover combination, on the other hand, provided a more accurate distance reading of 150cm, as opposed to the actual reading of 141cm. Additionally, at the 172° angle both man and woman’s bodies were detected by the sensor. 

For the squatting toilet, actual distance (158cm) was higher than the sitting toilet. The best angle for the red cover was 176°. At 178° angle, IR could detect a man and not a woman’s body. Other angles gave a very high sensor distance, which is not suitable for human body detection. The blue cover gave the same best angle which was 176°, but the sensor distance was higher than the red cover. Finally, the red and blue cover combination gave more accurate distance of up to 163cm from the actual reading. The 176° angle could also detect both man and woman’s bodies.

## Discussion

This project was carried out at a temperature of 30° C, which can affect the temperature of the sensor and as a result increase the sensor detection error. The formulation of percentage error is shown in the equation below.

$$\%\;\mathrm{Error}\;=\;\frac{\left|sensor\;distance-actual\;distance\right|}{actual\;distance}\;\times \;100$$

^10^

For sitting toilet, the lowest percentage error was 6.38%, which was obtained with the combination of red and blue plastic cover at 172° ( Table 1). This angle was the best suited for the human body for this type of toilet. The red cover gave the lowest percentage error (22%), which was good but not consistent. The distance for the red cover was challenging to calculate due to the fluctuations in the value. For the blue cover, percentage error was higher (30.5%) than the red cover. The blue cover deflects more IR light.

For squatting toilet, the lowest percentage error was 3.16%, which was obtained with the combination of red and blue plastic cover at 176° ( Table 2). This angle is the best suited for the human body for this type of toilet. The red cover gave the lowest percentage error (19.6%). Similar to the sitting toilet, measuring the distance was difficult due to the fluctuating value of the red cover. For the blue cover, percentage error was 10.76%, which was lower than the red cover.

In a similar study we used ultrasonic sound to detect the user’s presence in a smart toilet ^3^. This form of detection proved challenging as the sound wave was absorbed by the participant’s clothes, as a result the accurate distance could not be detected by the ultrasonic sound ^3^. Therefore, compared to our previous study, IR sensor is a more reliable detection system for smart toilets as it can precisely detect human presence in the toilet.

## Conclusions

This study utilizes an IR sensor with various angles and several different types of plastic covers to detect the user’s presence and distance from the smart toilet. Red cover and blue cover provide fluctuating distances for the sensor, which also impacts accuracy. The combination of blue and red plastic hides the sensor better, preventing the user from seeing it clearly while increasing accuracy. To get more reliable and precise results, the sensor voltage can be increased. This study has shown that IR sensor can detect human body with different postures more accurately while providing precise distance with the combination of the blue and red covers. This study can be further improved with implementation with various different materials which is more in industrial standard. 

### Ethics approval

The Research Ethics Committee (REC) of Multimedia University has granted ethical approval for this research with the approval number EA0592021. This study did not obtain participant personal details, therefore taking informed consent was not required.

## Data availability

### Underlying data

Figshare: An overview of infrared sensor's capability in Internet of Things implementation

 DOI: https://doi.org/10.6084/m9.figshare.16571331 ^9^

This project contains the following underlying data:

Data file. This file contains all the data that was generated from our analysis.

Data are available under the terms of the Creative Commons Zero "No rights reserved" data waiver ( CC0 1.0 Public domain dedication).

