# Supplementary material for: The accuracy of Infrared sensor detection in a smart toilet
Source: F1000Res. 2022 Mar 14;10:949. Originally published 2021 Sep 21. [Version 2] doi: 10.12688/f1000research.73086.2 (PMC11109674; doi:10.12688/f1000research.73086.2)
Supplement: Peer review history

# Reviewer report: Reviewer response for version 2

**DOI:** 10.5256/f1000research.122235.r178495

**Published:** 2024-05-11

**Authors:** Jin Zhou

**Affiliations:** 1 Duke University, Durham, North Carolina, USA

This paper provides an experimental study about the use of an IR sensor for human detection in a smart toilet. While the overall presentation is clear, certain aspects need clarification or improvement.

 1. The motivation of detecting a user's proximity to the smart toilet is not very clear. Can the authors elaborate on the practical applications of this functionality, such as touch-free flushing?

 2. The Introduction section includes two paragraphs detailing examples of IR sensor applications in different fields, which do not seem directly relevant to the paper's main topic. Condensing it might improve the overall flow.

 3. In the 'Raspberry Pi Programming Function' section, numbers used in the pseudocode need some explanations, are they linked to the specifics of the IR sensor used?

 4. In the ‘Results’ section, sensor distance seems to be always lager than actual distance. If possible, could we tune the parameters in the pseudocode to improve the performance?

 5. How many trials were conducted for each angle-color combination. If each combination was tested only once, could the authors comment on the reproducibility and reliability of the results?

Is the work clearly and accurately presented and does it cite the current literature?

Yes

If applicable, is the statistical analysis and its interpretation appropriate?

Partly

Are all the source data underlying the results available to ensure full reproducibility?

No source data required

Is the study design appropriate and is the work technically sound?

Partly

Are the conclusions drawn adequately supported by the results?

Partly

Are sufficient details of methods and analysis provided to allow replication by others?

Partly

Reviewer Expertise:

sensor array optimization; medical image analysis; machine learning

I confirm that I have read this submission and believe that I have an appropriate level of expertise to confirm that it is of an acceptable scientific standard, however I have significant reservations, as outlined above.

# Reviewer report: Reviewer response for version 2

**DOI:** 10.5256/f1000research.122235.r178516

**Published:** 2024-05-11

**Authors:** Cosmas Ifeanyi Nwakanma

**Affiliations:** 1 Kumoh National Institute of Technology, Gumi-si, Gyeongsangbuk-do, South Korea

A. The only missing link is the dearth of Citations and references. the reviewer will appreciate it if critical claims are supported with citations. Examples of such claims are:

 1. "The SHARP GP2Y0A710K0F is an IR distance sensor with an extra-long range of 100–500 cm, which is incredibly simple to use. As such, this IR sensor is a preferred option for this study compared to ultrasonic sensors".

 2. "To connect to the Raspberry Pi, the MCP 3008 chip is utilized as an analogue to digital converter".

 The critical question here is "What will happen if MCP 3008 is not used and how can readers know about this chip? Citations and explanations will help. 

 B. There seems to be too much "free advertisement" of products. For instance the mentions of "MCP 3008", and  "SHARP GP2Y0A710K0F". A general name of the product would have been better as readers might be given the impression that these results are not possible with other company products. 

 Check this reference if relevant

 Nwakanma, C.I et al, (2021 ^1^)

Is the work clearly and accurately presented and does it cite the current literature?

Partly

If applicable, is the statistical analysis and its interpretation appropriate?

Yes

Are all the source data underlying the results available to ensure full reproducibility?

Yes

Is the study design appropriate and is the work technically sound?

Yes

Are the conclusions drawn adequately supported by the results?

Yes

Are sufficient details of methods and analysis provided to allow replication by others?

Yes

Reviewer Expertise:

Internet of Things and Machine Learning for Smart Spaces.

I confirm that I have read this submission and believe that I have an appropriate level of expertise to confirm that it is of an acceptable scientific standard, however I have significant reservations, as outlined above.

## References

[1] : Detection and Classification of Human Activity for Emergency Response in Smart Factory Shop Floor. *Applied Sciences* .2021;11(8) : 10.3390/app11083662 10.3390/app11083662

# Reviewer report: Reviewer response for version 2

**DOI:** 10.5256/f1000research.122235.r178506

**Published:** 2024-05-11

**Authors:** Isaac Machorro-Cano

**Affiliations:** 1 Universidad del Papaloapan, Loma Bonita, Oaxaca, Mexico

The proposal presented an IR sensor with various angles and several different types of plastic covers to detect the user’s presence and distance from the smart toilet. The proposal presents an actual and interesting topic; however, the following aspects were identified:

 1.         In the introduction, it is suggested to give a broader context of the problems of the use of the IR sensor, especially to indicate the advantages of a correct functioning in terms of people's health. In this context, it is suggested to mention worldwide statistical data of this problem or opportunity for innovation using the IoT. Indicate the advantages of using IoT in this context and its importance for innovation. Indicate and clearly highlight the scientific contribution of the proposal.

 2. It is noted that only three papers related to the proposal are indicated, but it is suggested to increase the search for related papers of the last 7 years to identify those papers that are most related to the proposal, especially in the context of research and it is necessary to perform a comparative analysis and indicate the main differences of the proposal with respect to the related papers identified to determine and clarify the novelty of the work presented.

 3. Why is it important to detect the human toilet position?, why use the IR sensor and not another sensor?, what other sensors could be used?, what happens if the person does not have a good defecation posture?.

 4.         It is observed that a section of obtained results is presented, however, a table of all the obtained results is not indicated in which it is possible to perceive the cases of correct detection of the IR sensor. Also, if the sensor detects a bad posture of the human being, is it indicated to the user?, is it verified that he/she is in the correct posture?, and when entering the toilet is the correct posture visually indicated to the user?

 5.         In the discussion, the authors are expected to discuss at length the findings and challenges identified, as well as their impressions and comments based on the results obtained. Reaffirm and discuss the scientific contribution.

 6.         In the conclusions, the main conclusion of the scientific contribution is not indicated, and the future work to be done is not indicated.

Is the work clearly and accurately presented and does it cite the current literature?

Partly

If applicable, is the statistical analysis and its interpretation appropriate?

Not applicable

Are all the source data underlying the results available to ensure full reproducibility?

Partly

Is the study design appropriate and is the work technically sound?

Partly

Are the conclusions drawn adequately supported by the results?

Partly

Are sufficient details of methods and analysis provided to allow replication by others?

Yes

Reviewer Expertise:

IoT

I confirm that I have read this submission and believe that I have an appropriate level of expertise to state that I do not consider it to be of an acceptable scientific standard, for reasons outlined above.

# Reviewer report: Reviewer response for version 2

**DOI:** 10.5256/f1000research.122235.r127394

**Published:** 2022-03-28

**Authors:** Mira Kania Sabariah

**Affiliations:** 1 School of Computing, Telkom University, Bandung, Indonesia

I have received all the revisions, so I declare this article has been approved

Is the work clearly and accurately presented and does it cite the current literature?

Yes

If applicable, is the statistical analysis and its interpretation appropriate?

Yes

Are all the source data underlying the results available to ensure full reproducibility?

No source data required

Is the study design appropriate and is the work technically sound?

Yes

Are the conclusions drawn adequately supported by the results?

Partly

Are sufficient details of methods and analysis provided to allow replication by others?

No

Reviewer Expertise:

Software Engineering

I confirm that I have read this submission and believe that I have an appropriate level of expertise to confirm that it is of an acceptable scientific standard.

# Reviewer report: Reviewer response for version 1

**DOI:** 10.5256/f1000research.76710.r124303

**Published:** 2022-03-04

**Authors:** Mira Kania Sabariah

**Affiliations:** 1 School of Computing, Telkom University, Bandung, Indonesia

- The research idea is good.
- There needs to be justification for the number of participants which only involved 12 participants. According to statistics, in determining the number of samples, it is necessary to pay attention to the number of populations or to follow a minimum limit of 30.
- The research steps have not been presented in the paper.
- The scenarios and instruments/tools of the experiments carried out were not well explained in the paper.
- The reason for choosing the colour and combination of plastic covers should be explained.
- The factors that become the limitations of the research carried out must be conveyed to the conclusion.

Is the work clearly and accurately presented and does it cite the current literature?

Yes

If applicable, is the statistical analysis and its interpretation appropriate?

Yes

Are all the source data underlying the results available to ensure full reproducibility?

No source data required

Is the study design appropriate and is the work technically sound?

Yes

Are the conclusions drawn adequately supported by the results?

Partly

Are sufficient details of methods and analysis provided to allow replication by others?

No

Reviewer Expertise:

Software Engineering

I confirm that I have read this submission and believe that I have an appropriate level of expertise to confirm that it is of an acceptable scientific standard, however I have significant reservations, as outlined above.
